# Probing the Effect of Alloying Elements on the Interfacial Segregation Behavior and Electronic Properties of Mg/Ti Interface via First-Principles Calculations

**DOI:** 10.3390/molecules29174138

**Published:** 2024-08-31

**Authors:** Yunxuan Zhou, Hao Lv, Tao Chen, Shijun Tong, Yulin Zhang, Bin Wang, Jun Tan, Xianhua Chen, Fusheng Pan

**Affiliations:** 1 Lanxi Magnesium Materials Research Institute, Lanxi 321100, China; yunxuanzhou93@gmail.com (Y.Z.); 18818276064@163.com (S.T.); zhangyulin@pku.edu.cn (Y.Z.); zjnuwangbin@163.com (B.W.); xhchen@cqu.edu.cn (X.C.); fspan@cqu.edu.cn (F.P.); 2 National Engineering Research Center for Magnesium Alloys, College of Materials Science and Engineering, Chongqing University, Chongqing 400044, China; aicaodafu@sina.com

**Keywords:** Mg/Ti interface, elastic anisotropy, interface segregation, electronic properties, alloying elements

## Abstract

The interface connects the reinforced phase and the matrix of materials, with its microstructure and interfacial configurations directly impacting the overall performance of composites. In this study, utilizing seven atomic layers of Mg(0001) and Ti(0001) surface slab models, four different Mg(0001)/Ti(0001) interfaces with varying atomic stacking configurations were constructed. The calculated interface adhesion energy and electronic bonding information of the Mg(0001)/Ti(0001) interface reveal that the HCP2 interface configuration exhibits the best stability. Moreover, Si, Ca, Sc, V, Cr, Mn, Fe, Cu, Zn, Y, Zr, Nb, Mo, Sn, La, Ce, Nd, and Gd elements are introduced into the Mg/Ti interface layer or interfacial sublayer of the HCP2 configurations, and their interfacial segregation behavior is investigated systematically. The results indicate that Gd atom doping in the Mg(0001)/Ti(0001) interface exhibits the smallest heat of segregation, with a value of −5.83 eV. However, Ca and La atom doping in the Mg(0001)/Ti(0001) interface show larger heat of segregation, with values of 0.84 and 0.63 eV, respectively. This implies that the Gd atom exhibits a higher propensity to segregate at the interface, whereas the Ca and La atoms are less inclined to segregate. Moreover, the electronic density is thoroughly analyzed to elucidate the interfacial segregation behavior. The research findings presented in this paper offer valuable guidance and insights for designing the composition of magnesium-based composites.

## 1. Introduction

Magnesium (Mg) alloys are referred to as the “green engineering materials of the 21st century” owing to their numerous outstanding properties, including superior specific strength, specific stiffness, excellent machinability, and environmental friendliness [1,2,3,4,5]. Consequently, they have garnered considerable attention and hold significant potential for applications in high-performance automotive, aerospace, hydrogen storage, and 3C industries [6,7,8,9,10]. However, in comparison to traditional materials such as aluminum alloys, Mg alloys feature a hexagonal close-packed (HCP) crystal structure with a limited number of slip systems, leading to a limited plastic deformation capability [11]; they also face challenges such as low modulus and poor formability. This limitation significantly impedes the industrial utilization of Mg alloys [12]. Developing and applying high-performance Mg alloys is crucial for bolstering and advancing the Mg alloy industry, as well as boosting manufacturing competitiveness.

Scholars in pertinent research have concentrated on enhancing the overall performance of Mg alloy materials through strategies like compositional design, alloying with trace elements, and employing heat treatment processes [13]. The in situ formation or the incorporation of particles is also a potent method for boosting the strength and elastic modulus (stiffness) of Mg alloys [14,15]. This involves incorporating a second phase with high modulus, good toughness, high thermal stability, and excellent dynamic stability to improve the strength, modulus, and stability of Mg alloys. Whether it is externally added particles or in situ formed second phases, interfaces are formed between the enhancement phase and the Mg matrix, and the microstructure and interface bonding strength significantly affect the performance of Mg-based composite materials [16]. Ceramic particle reinforcements (like SiC, Al_2_O_3_, B_4_C, TiC, TiB_2_, etc.) are widely used in Mg-based composite materials [17,18,19]. Ding et al. [20] prepared CNTs@SiC/Mg-6Zn composites through chemical vapor deposition, semi-solid stirring combined with ultrasonic treatment, and hot extrusion deformation. Their finding revealed that while the yield strength of the composite rose from 154 MPa to 218 MPa, its room-temperature fracture elongation decreased from 15.6% to 6.1%. Although the addition of ceramic particles can enhance the corrosion resistance, strength, wear resistance, and modulus of Mg-based composite materials, it can also lead to a decrease in material fracture toughness (some composites have an elongation below 1%) due to their low plastic formability and poor thermal physical compatibility at the interface. Moreover, such an utterly incoherent interface easily generates stress concentration, leading to the initiation of microcracks. On the other hand, the ceramic reinforcement itself is a brittle phase, resulting in a low plasticity in Mg-based composites, making industrial application of such materials quite challenging. Therefore, finding reinforcements with high plastic deformation capability that closely match the Mg’s crystallographic and physical characteristics is a potential method for enhancing the strength, plasticity, and modulus of Mg-based composites.

Numerous experimental results also demonstrate that titanium (Ti) can effectively enhance the strength and plasticity of Mg-based composite materials [21]. Perez et al. [22] prepared Mg-10(vol.%)Ti composite materials using powder metallurgy forming combined with hot extrusion deformation. The tensile strength at room temperature was measured to be 160 MPa, with an elongation of 8%. Furthermore, Wu et al. [23] developed Ti particle-reinforced Mg-based composite materials, where Ti particles form interfaces with the Mg alloy matrix featuring an elementally graded distribution structure. This composite material achieves a tensile strength of 327 MPa and an elongation of 20.4%. In addition, some researchers have utilized first-principles calculations to probe the interfacial properties between ceramic reinforcements (or second phases) and the Mg matrix (such as Mg/TiC, Mg/AlN, Mg/ZnO, Mg/Al_2_MgC_2_, Mg/Mg_2_Sn, Mg/Al_4_C_3_, Mg/TiB_2_, Mg/ZrB_2_, Mg/Ti_2_AlC, Mg/Al_2_MgC_2_, Mg/Al_2_CO, etc.) [24,25,26,27,28,29,30,31,32,33]. Furthermore, the dynamic stability of some second phases was assessed through phonon spectrum analysis. The calculation results of elastic properties indicate these phases possess a high Young’s modulus, suggesting their potential as reinforcing phases. Moreover, some researchers have studied the stability and elastic properties of binary (60.51 GPa for Young’s modulus in Mg_2_Sn phase, 71–94 GPa for Young’s modulus in Mg_17_Al_12_ phase) or ternary phases (72.15–98.27 GPa for Young’s modulus in Mg_32_(Al,Zn)_49_ phase with different Al:Zn atomic ratio) in magnesium alloys or Mg-based composites [34,35,36]. There are also studies on Mg matrix composites reinforced with heterogeneous metals. For example, the adhesion strength and interfacial bonding of various Mg/X (X = Ti, Zr, Hf, V, Nd, Cr, Mo, Mn, and Fe) interface configurations were investigated by Bao et al. [37]. Relevant studies have shown that Ti, with its HCP structure matching that of Mg, can form a coherent Mg/Ti interface, thereby contributing to the enhancement of the Mg alloys’ comprehensive performance. Additionally, Ti and Mg do not mix at equilibrium, indicating that there is no reaction between Ti and Mg elements, thereby inhibiting the formation of brittle intermetallic compounds at the Mg-Ti interface. This formable Ti forms a strong interface bond with Mg and exhibits fewer internal defects, thus better enhancing the material’s properties. However, the segregation behavior of common alloying elements in Mg/Ti interfaces is still poorly understood. This work employs the first-principles calculations method to establish various interface configurations between the heterogeneous metal Ti and Mg matrix. Additionally, low-cost alloying elements and rare earth elements are introduced at the interface to investigate their segregation behavior. This research provides valuable insights into the development of heterogeneous metal-reinforced Mg-based composite materials.

## 2. Results and Discussion

### 2.1. Bulk and Surface Properties

**Figure 1:**
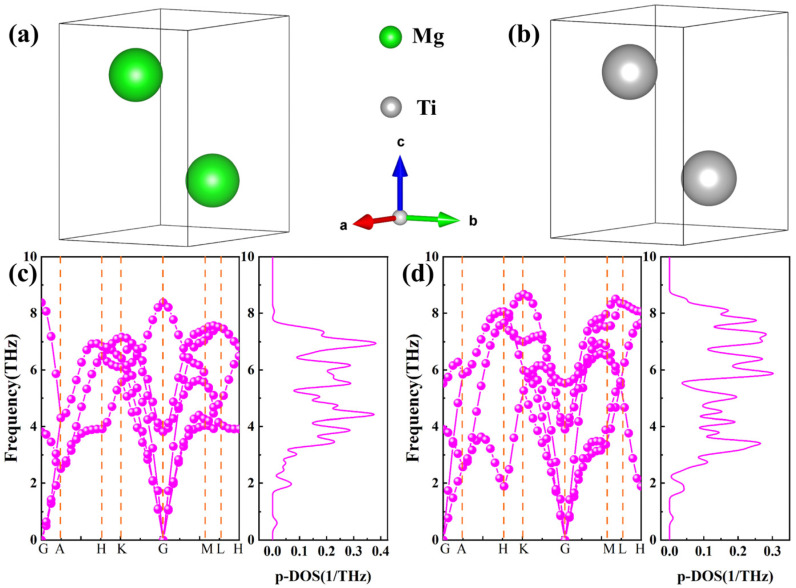
(**a**,**b**) The equilibrium crystal structures after geometric optimization of Mg and Ti, (**c**,**d**) Phonon spectra and phonon density of state of Mg and Ti, where green and gray spheres represent Mg and Ti atoms, respectively.

The equilibrium crystal structures after geometric optimization of pure Mg and Ti are depicted in Figure 1a,b, and the green and gray spheres denote Mg and Ti atoms, respectively. The Mg has the HCP structure with a space group of P63/mmc, featuring only one standard Wyckoff site, namely 2c (0.67, 0.33, 0.75). The optimized lattice parameters of Mg are approximately a = b = 3.19 Å and c = 5.29 Å, which closely match experimental values (a = b = 3.210 Å and c = 5.211 Å; a = b = 3.209 Å, c = 5.171 Å) and other computational results (a = b = 3.159~3.228 Å and c = 5.073~5.186 Å) [31,38,39]. For titanium (Ti), generally, there are two allotropes of α-phase (with HCP structure) and the β-phase (with BCC structure), and the critical temperature of the phase transition is approximately 882.5 °C [40]. Considering the actual service of Mg alloys, the temperature is below 882.5 °C; therefore, α-Ti is chosen in this work. Moreover, α-Ti belongs to the P63/mmc space group, with theoretical lattice constants of a = b = 2.94 Å, c = 4.64 Å, α = β = 90°, and γ = 120° [41], where the Wyckoff position for Ti atoms is 2c(1/3, 2/3, 1/4). The calculated lattice parameters of this phase are a = b = 2.91 Å, c = 4.59 Å, α = β = 90°, and γ = 120°, respectively. It can be observed that the lattice constants obtained through GGA + PBE calculations exhibit a variation of approximately 1.03% compared to the theoretical values, showing the validity of the selected parameters. In addition, the calculated c/a ratio for α-Ti is approximately 1.57, while for Mg, it is 1.65, indicating that α-Ti has a better formability than Mg [42,43], which is advantageous for enhancing the formability of Mg alloys.

Phonon calculations of Mg and Ti offer a criterion for the stability of crystals. Specifically, in any high-symmetry dispersion, the absence of imaginary frequencies serves as an indicator that the crystal structure is dynamically stable. The calculated phonon spectra and phonon density of states for pure Mg and Ti with HCP structures are illustrated in Figure 1c,d, with the phonon spectrum path being *G*-*A*-*H*-*K*-*G*-*M*-*L*-*H*. It can be observed from Figure 1 that the long-range coulomb interaction in the phonon spectrum results in higher frequencies for the longitudinal optical branches compared to the transverse optical branches. In other words, at the high-symmetry point *G*, the three phonon spectra with relatively lower frequencies correspond to acoustic phonon branches, while the spectra with relatively higher frequencies correspond to optical phonon branches. The phonon spectra, furthermore, do not contain imaginary frequencies at any high-symmetry point, showing that both Mg and Ti are dynamically stable. Additionally, the calculated phonon density of states, as presented in Figure 1c,d, reveals that the phonon spectra are primarily distributed within the frequency range of 2–8 THz. Moreover, the distribution of phonon spectra is similar, with only slight variations in trends around local high-symmetry points. For example, at the *K* high-symmetry point, the frequencies of Ti are notably higher than those of Mg. Noticeably, the vibrational spectrum of the phonon density of states for Mg atoms is higher than that for Ti atoms due to the lower mass of Mg atoms compared to Ti atoms, with both falling below 0.4 1/THz.

**Figure 2:**
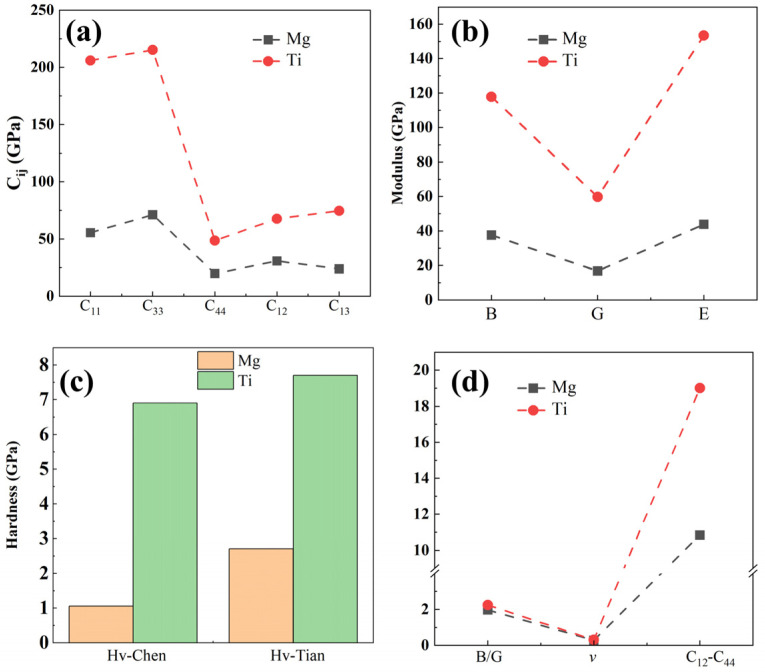
The mechanical properties of Mg and Ti calculated using first-principles calculations are (**a**) elastic constants, (**b**) elastic modulus, including bulk modulus (*B*), shear modulus (*G*), and Young’s modulus (*E*), (**c**) predicted hardness by the Chen’s and Tian’s models, and (**d**) brittleness and toughness.

To investigate the mechanical properties of pure Mg and Ti, we employed the stress–strain method to predict the elastic stiffness matrix for each phase [44,45]. Subsequently, using the Voigt–Ruess–Hill approximation method [46], we derive the elastic constants (Cij), elastic modulus, and hardness, as depicted in Figure 2a–c. It is important to note that the formulas for calculating elastic modulus, Poisson’s ratio, and hardness can be found in Bao et al.’s work [47]. Noticeably, both the elastic constants and elastic modulus, as well as the hardness of Ti, are significantly greater than those of Mg, showing it has a strengthening effect in Mg alloys. The calculated Young’s modulus of Ti is ~153.4 GPa, significantly higher than Mg’s 43.9 GPa (close to the experimental value of ~45 GPa [48]), and higher than other binary or ternary phases (like Al_2_Gd (144.2 GPa), Mg_2_Gd_5_ (74.8 GPa), Mg_24_Y_5_ (66.6 GPa), MgZn (79.1 GPa), Al_2_Ca (94.34 GPa), β′-Mg_7_Tb (57.1 GPa), Al_2_Li_3_ (125.4 GPa), Al_3_CuCe (82.2 GPa), and Mg_3_(MnAl_9_)_2_ (125.6 GPa) [49,50,51,52,53], indicating that Ti plays a positive role in enhancing the modulus of Mg alloys. The C_11_ value for Ti is the highest, with approximately 205.9 GPa, suggesting that Ti exhibits better incompressibility than Mg (~55.4 GPa for C_11_). This implies that it is more resistant to compression along the a-axis (ε_11_ direction) under uniaxial stress. The smaller C_11_ of Mg (approximately 19.9 GPa) indicates that Mg is more susceptible to shear deformation on the (100) crystal plane than Ti. Moreover, the predicted hardness values by Chen’s and Tian’s models are displayed in Figure 2c. Furthermore, the hardness of Ti predicted by both models is greater than that of Mg. According to Tian’s model, the hardness values of Ti and Mg are 7.72 and 2.71 GPa, respectively, representing a difference of about three times. The Poisson’s ratio, Cauchy pressure, and bulk modulus to shear modulus (*B*/*G*) ratio can all serve to assess the brittleness and toughness of materials. Generally, the critical values for brittleness and toughness of the Poisson ratio and *B*/*G* ratio are 0.26 and 1.75, respectively. Observations from Figure 2d reveal that the *B*/*G* and Poisson’s ratios (*v*) of pure Ti are marginally higher than those of pure Mg, suggesting a slightly superior plasticity in Ti compared to Mg. Nonetheless, both Ti and Mg exhibit calculated values around 0.30, indicating similar metallic characteristics. Additionally, using the Cauchy pressure (C_12_–C_44_) to assess their ductility, it is evident that the Cauchy pressure of Ti is much greater than that of Mg, indicating that Ti has a better plastic deformation capability.

**Figure 3:**
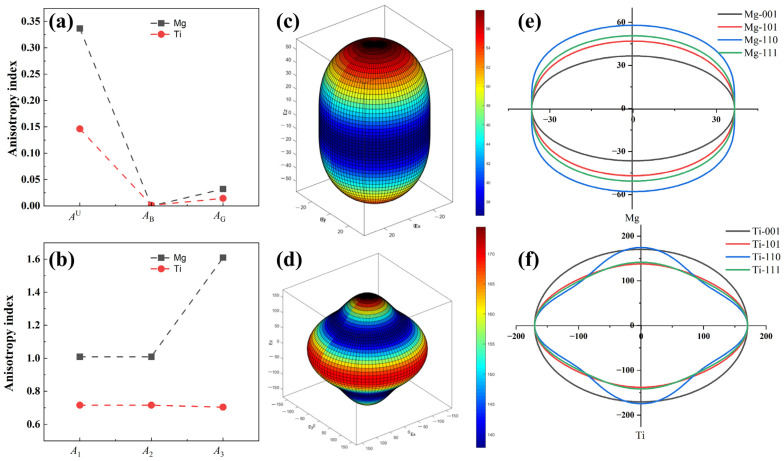
The anisotropy of Mg and Ti crystal structures is (**a**) *A*^U^, *A*_B_, *A*_G_, (**b**) *A*_1_, *A*_2_, and *A*_3_, three-dimensional (3D) surface plots and two-dimensional (2D) projections with different planes of Young’s modulus for Mg (**c**,**e**) and Ti (**d**,**f**) crystal structures, and the unit for Young’s modulus in (**c**–**f**) is GPa.

The anisotropy indices of Mg and Ti, obtained through DFT calculations, are depicted in Figure 3, and the specific elastic anisotropy calculation formulas can be described in the works of Ranganathan et al. [54]. The calculated anisotropy indices *A*^U^, *A*_B_, and *A*_G_, and the shear anisotropy indices (*A*_1_, *A*_2_, and *A*_3_) are depicted in Figure 3a,b. Analysis from Figure 3a,b reveal that Mg exhibits greater anisotropy than Ti, with *A*^U^ values of 0.34 and 0.15 for Mg and Ti, respectively. The anisotropy of Mg is approximately twice that of Ti. The shear anisotropy factor values for Mg, *A*_1_, *A*_2_, and *A*_3_ are 1.01, 1.01, and 1.61, respectively, which are greater than Ti’s shear anisotropy indices (*A*_1_ = *A*_2_ = 0.72 and *A*_3_ = 0.71), indicating Mg exhibits a more noticeable shear anisotropy. The three-dimensional (3D) surface plots of Young’s modulus anisotropy for Mg and Ti, moreover, are represented in Figure 3c,d. It can be observed from the figures that the 3D plots of Young’s modulus of Mg and Ti deviate from sphericity, indicating their anisotropic. Noticeably, 3D plots of Young’s modulus for Mg exhibit more deviation from sphericity compared to Ti, suggesting that the anisotropy of Young’s modulus for Mg is stronger than that of Ti, which aligns with the results of our calculated anisotropy index of *A*^U^. In addition, Figure 3e,f illustrate the two-dimensional (2D) projections of Young’s modulus anisotropy for Mg and Ti. Overall, the 2D of Ti’s Young’s modulus exhibits smaller differences in different directions compared to Mg’s projection. This indicates that Ti has a lower anisotropy. The anisotropic Young’s modulus results mentioned above play a critical role in designing the texture of Mg alloys. They have important implications for the design of laminated composite panels or Mg-based composite materials reinforced with heterogeneous metals.

**Figure 4:**
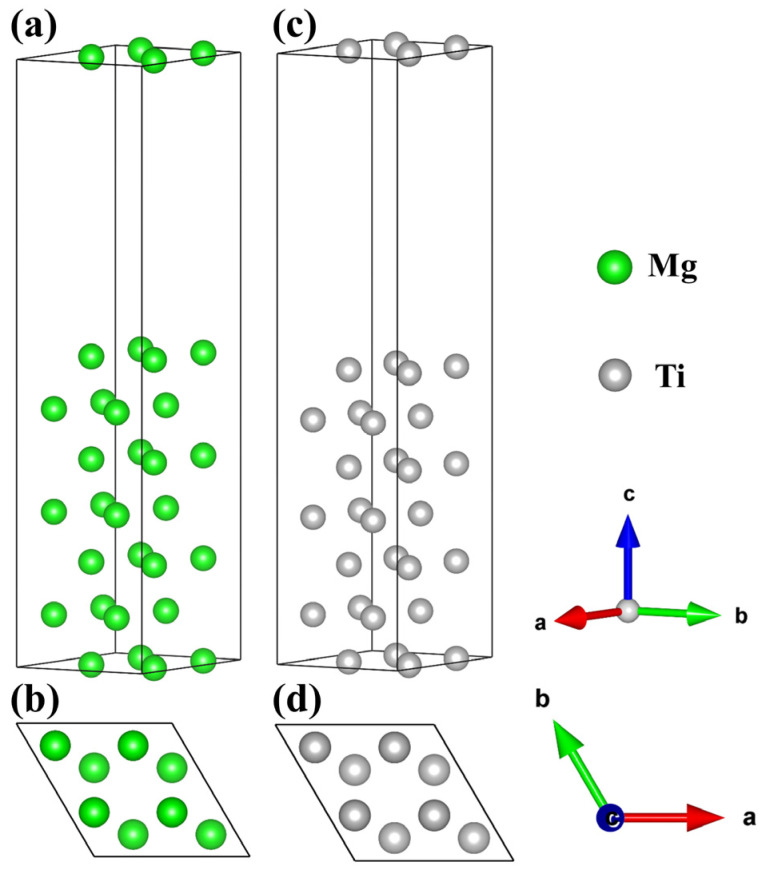
The surface configurations of Mg and Ti. Top and side views of the Mg(0001) (**a**,**b**) and Ti(0001) (**c**,**d**) surfaces.

Before performing interface calculations, it is necessary to relax and compute the Mg and Ti surfaces. Generally, the greater the number of atoms in the surface model, signifying more layers of atoms, the closer the properties exhibited by the surface will be to those of the bulk material. Additionally, when constructing the model, it is essential to consider the computational capability of the server and ensure that both surfaces approximate the properties of the bulk material. Considering the space group symmetry and lattice matching of Mg and Ti, the (0001) surfaces of Mg and Ti are cleaved based on bulk phases, and a 2 × 2 supercell is created. Previous literature suggests that a slab model consisting of seven atomic layers of Mg is adequate to portray bulk-like properties. Similarly, a 2 × 2 supercell model with seven atomic layers of Ti is also deemed sufficient to capture bulk-like properties [55]. The Mg(0001) and Ti(0001) surface configurations with top and side views are displayed in Figure 4. Noticeably, the Mg(0001) and Ti(0001) surface slab models consist of 28 Mg atoms and 28 Ti atoms, with each layer containing either 4 Mg atoms or 4 Ti atoms. The relaxed lattice constants for the Mg(0001) slab model are as follows: a = b = 6.45 Å, c = 30.45 Å; α = β = 90°, γ = 120°. Similarly, for the Ti(0001) slab model, the relaxed lattice constants are: a = b = 5.92 Å, c = 28.74 Å; α = β = 90°, γ = 120°. Notably, both structures demonstrate matching in lattice parameters and angles, facilitating the establishment of interface models.

### 2.2. Properties of the Mg/Ti Interface

#### 2.2.1. Interfacial Configuration

**Figure 5:**
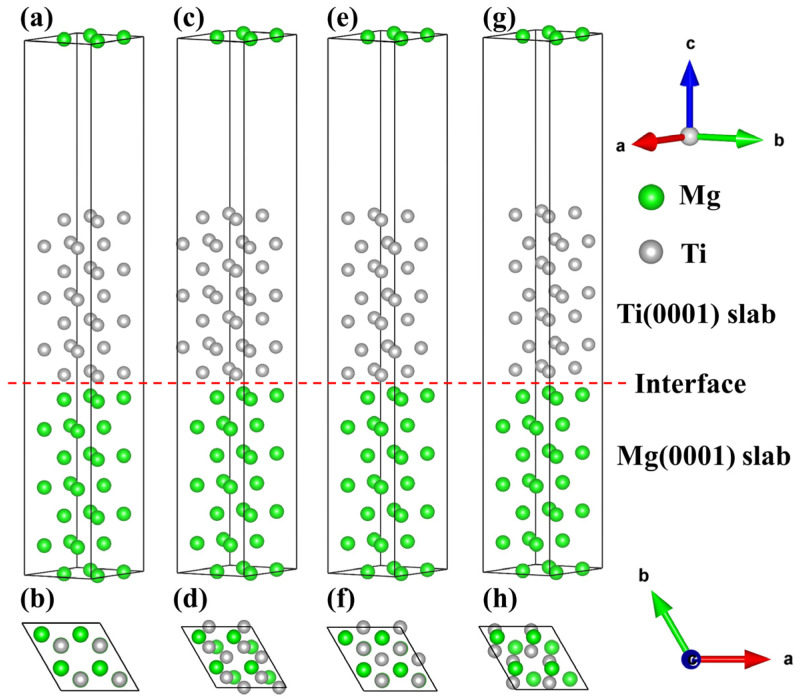
The interfacial configurations of the Mg(0001)/Ti(0001) interface with different atomic stacking sequences, (**a**,**b**) OT configuration, (**c**,**d**) MT configuration, (**e**,**f**) HCP1 configuration, and (**g**,**h**) HCP2 configuration.

Establishing the interface model requires consideration of the phase matching at the interface, and the mismatch of lattice parameters and angles must be calculated [56,57,58]. Furthermore, Ti with an HCP crystal structure possesses a higher Young’s modulus of approximately 153.4 GPa and a lower anisotropy, which can serve as a reinforcement material. Based on the dynamic stability of the bulk material, we optimize the lattice constants of the bulk Mg and Ti and establish the interface structure based on the lattice constants of the bulk material. Exactly, the lower the mismatch at the interface is, the better the interface matching is [59]. Through a comparison of the lattice constants, surface, and interface areas of Mg and Ti, it was found that the interfacial mismatch was less than 5%, showing good interface matching [60,61]. There are four different configurations in the Mg/Ti interface, namely, OT, MT, HCP1, and HCP2, as shown in Figure 5. Specifically, the OT configuration indicates that Mg atoms at the interface are directly positioned on top of the first layer of Ti(0001) atoms. The MT configuration depicts Mg atoms at the interface located above the midpoint of the atomic connections in the first layer of Ti(0001) atoms. The HCP1 configuration shows Mg atoms at the interface situated in the polyhedral vacancies of the first layer of Ti(0001) atoms. Lastly, the HCP2 configuration illustrates Ti atoms at the interface occupying the polyhedral vacancies of the first layer of Mg(0001) atoms. The interface configurations of Mg(0001)/Ti(0001) with different atomic stacking sequences are illustrated in Figure 5. Here, (a, c, e, g) represent the main views of the interface stacking models. In contrast, (b, d, f, g) depict the top views of the interface stacking models. Additionally, the red dashed line represents the interface between Mg and Ti, where the area above the red line indicates the Ti(0001) surface, and the area below the red line represents the Mg(0001) surface.

**Figure 6:**
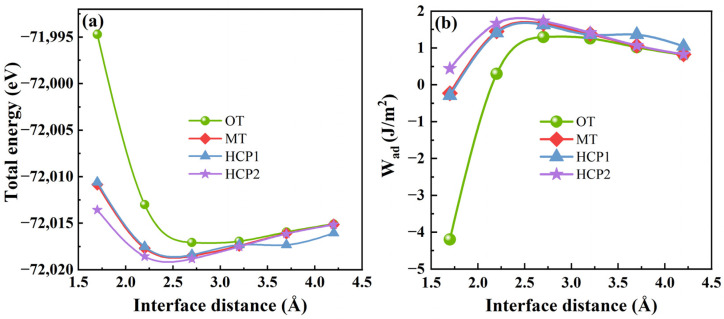
The interfacial total energy (**a**) and the ideal interface adhesion energy (**b**) of Mg(0001)/Ti(0001) interface with different atomic stacking sequences.

The interface adhesion energy (*W*_ad_) is the reversible work per unit area required to separate the Mg(0001)/Ti(0001) interface between condensed phases Mg(0001) and Ti(0001) to generate two free surfaces, which denotes [62,63]:

(1) $$W_{ad}=(E_{Mg}^{slab}+E_{Ti}^{slab}-E_{Mg/Ti}^{\mathrm{int}erface})/A_{i}$$

where the $E_{Mg}^{slab}$ and $E_{Ti}^{slab}$ are total energies of a seven-layer Mg(0001) and seven-layer Ti(0001) free surface, $E_{Mg/Ti}^{\mathrm{int}erface}$ is the total energy of interface, and *A_i_* represents the interface area of Mg(0001)/Ti(0001) interface. In general, the interfacial distance significantly influences the interface performance. Hence, it is essential to conduct tests to evaluate the interfacial distance [24,64]. On the other hand, the purpose of testing the interface distance is to understand the approximate bonding length, which can be expressed by the following formula:

(2) $$W_{ad}(d)=-W_{ad}\left[1+\frac{\left(d-d_{0}\right)}{l}\right]\exp\left[-\frac{\left(d-d_{0}\right)}{l}\right]$$

where *W*_ad_ is the ideal interface adhesion energy of the Mg(0001)/Ti(0001) interface. *d*_0_ and *d* represent the equilibrium interface separate value and the interfacial separation distance between the Mg(0001) and Ti(0001) slabs, and *l* is a Thomas–Fermi screening length. The interface total energy and the ideal interface adhesion energy (*W*_ad_) for various stacking models of Mg(0001)/Ti(0001) fluctuate with the separation distance of the Mg/Ti interface, as described in Figure 6. It is clear that the total interfacial energy of various atomic interface configurations of Mg(0001) and Ti(0001) decreases as the interface distance increases. Beyond a separation distance of 2.5 Å, the interfacial total energy tends to level off. Due to the energies of Mg(0001) and Ti(0001) surfaces, the area *A_i_* remains constant, with only the total energy of the Mg/Ti interface with different atomic configurations varying. As a result, Figure 6a,b exhibit similar shapes of the curves. Moreover, the OT model’s interfacial total energy variation is relatively larger than the other configurations. The *W*_ad_ exhibits an opposite trend as the interface distance increases. Initially, *W*_ad_ increases with the interface distance and then stabilizes within the 1.5 to 4.5 Å separation distance between the two rigid free surfaces. Therefore, the interface distance can be around 2.5 Å, according to the analysis of Figure 6. Additionally, based on the predicted interface distance, the lattice parameters a, b, and the three angles of the interface were fixed while atoms were relaxed. The interfacial properties and the effect of alloying elements on the interface were then calculated. 

#### 2.2.2. Interfacial Segregation Behavior and Interfacial Adhesion Work

**Figure 7:**
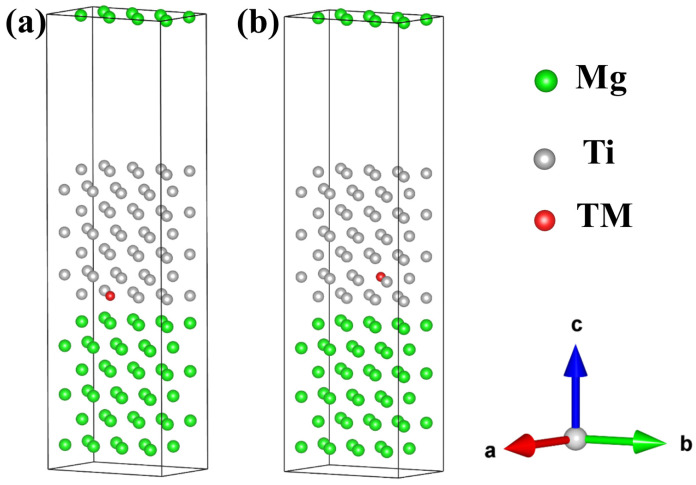
The interfacial configuration of alloy element doping at the Mg/Ti interface, (**a**) doping at the interface Ti layer, and (**b**) doping at the interface Ti sub-surface layer.

To investigate the influence of different alloying elements on interface properties and consider the solubility of trace alloying elements, we further expanded the aforementioned models using a 2 × 1 supercell method, achieving an approximate alloy concentration of 1%. Here, the most stable Mg(0001)/Ti(0001) interface with HCP2 configuration is chosen to investigate the influence of different alloying elements on its interfacial segregation behavior and stability. In the doped model, the total number of atoms is 112, including 56 Mg atoms, 55 Ti atoms, and one alloying element TM commonly found in Mg alloys (TM = Si, Ca, Sc, V, Cr, Mn, Fe, Cu, Zn, Y, Zr, Nb, Mo, Sn, La, Ce, Nd, and Gd), as shown in Figure 7, in which the green and gray spheres represent Mg and Ti atoms, respectively, and the red sphere represents the alloying atom TM. Based on the Wyckoff positions of Ti and Mg atoms in the bulk phase, it can be inferred that Ti or Mg atoms in the Mg(0001)/Ti(0001) interface models are equivalent. In the pure Mg(0001)/Ti(0001) interface model, one Ti atom is substituted by an alloying element TM, creating a doped model. Since the alloy concentration is approximately 1%, corresponding to a dilute model, its influence on lattice constants is negligible. Consequently, the interface distance is also approximately 2.5 Å.

The heat of segregation is an important indicator for characterizing the preference of alloying atom positions. It can also characterize the difficulty of segregation of atoms at a specific position in the interface models. The heat of segregation (Δ*E*_seg_) typically reflects the difficulty of alloy element segregation at the interface, as shown in Formula (3) [65,66,67]:

(3) $$\Delta E_{seg}=\frac{1}{n}\left(E_{Mg/Ti-TM}^{\mathrm{int}erface}-E_{Mg/Ti}^{\mathrm{int}erface}+n\mu_{Ti}-n\mu_{TM}\right)$$

**Figure 8:**
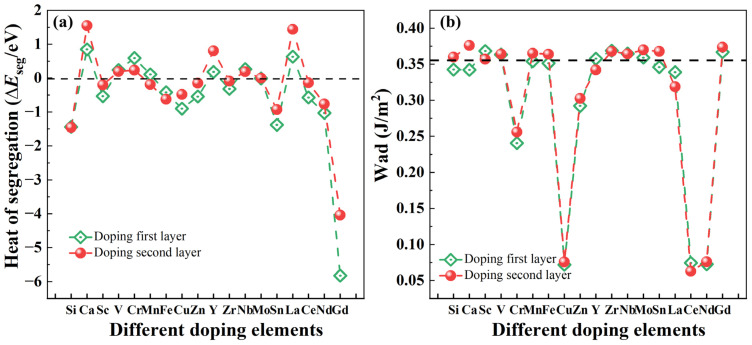
The heat of segregation (Δ*E*_seg_) (**a**) and work of adhesion (*W*_ad_) (b) of Mg(0001)/Ti(0001) interface with different alloy elements doping the first layer and the sub-surface layer of Ti atoms, and the dashed lines in (**b**) represent the *W*_ad_ of pure Mg/Ti interface.

In Formula (3), $E_{Mg/Ti-TM}^{\mathrm{int}erface}$ is the total energy after adding the alloying element on the interface Ti layer and Ti sub-surface layer of the Mg/Ti interface, $E_{Mg/Ti}^{\mathrm{int}erface}$ represents the total energy of the pure interface, $\mu_{Ti}$ and $\mu_{TM}$ represent the chemical potential of Ti and alloying element, which satisfy the following relationship [68,69]: $\mu_{TM}=E_{TM}^{bulk}/n_{x}$, and n represents the number of alloying element. Since we are considering only one alloying atom, n = 1 in this case. In general, a positive Δ*E*_seg_ suggests that the alloying element is less likely to segregate at the interface, while a negative Δ*E*_seg_ indicates a higher likelihood of segregation [70,71,72]. To investigate the interfacial segregation behavior of various alloying elements at the Mg(0001)/Ti(0001) interface, an alloy atom should be introduced into either the Ti layer or Ti sub-surface layer at the interface. Figure 8a depicts the calculated Δ*E*_seg_ values for various alloying elements at the interface. The findings reveal notable disparities in the heat of segregation, implying that these elements have the capability to segregate at the interface and impact its characteristics, even at low concentrations. Among them, after doping Gd atoms into the first layer of Ti atoms for the Mg(0001)/Ti(0001) interface, which exhibits the minimum Δ*E*_seg_ of approximately −5.83 eV. This suggests that, compared to other elements, Gd is most likely to segregate at this position. However, after doping Ca and La atoms into the first layer of Ti atoms, the interface exhibits larger Δ*E*_seg_ with values of 0.84 eV and 0.63 eV, respectively. This indicates that, compared to other alloying atoms, Ca and La atoms are less likely to segregate at the interface. Additionally, after doping alloying elements such as V, Cr, Mn, and Nb, the Δ*E*_seg_ values are positive, suggesting a more incredible difficulty in segregation at the interface. The heat of segregation of interface layer Ti atoms after doping different alloying atoms at the Mg(0001)/Ti(0001) interface follows the following order: Gd > Si > Sn > Nd > Cu > Ce > Zn > Sc > Fe > Zr > Mo > Mn > Y > V > Nb > Cr > La > Ca. Similarly, we have also calculated the Δ*E*_seg_ of the second outermost layer Ti atoms at the Mg(0001)/Ti(0001) interface following the doping of various alloying elements. The outcomes mirror those observed for doping at the interface layer Ti atoms, demonstrating the following order: Gd > Si > Sn > Nd > Fe > Cu > Sc > Mn > Zn > Ce > Zr > Mo > Nb > V > Cr > Y > La > Ca. Comparing the Δ*E*_seg_ values at the positions of the first or second outermost layer Ti atoms, it is evident that Gd, Si, Sn, and Nd are prone to segregation, whereas alloying elements like Ca, La, Cr, and V are less likely to undergo segregation. The segregation behavior of the aforementioned alloying elements at the Mg/Ti interface provides valuable guidance for designing and selecting heterogeneous metal Ti-reinforced Mg-based composite materials.

After doping alloying elements to the Mg/Ti interface, the *W*_ad_ can be used as:

(4) $$W_{ad}=(E_{Mg}^{slab}+E_{Ti-TM}^{slab}-E_{Mg/Ti-TM}^{\mathrm{int}erface})/A_{i}$$

where the $E_{Ti-TM}^{slab}$ is total energy of a seven-layer Ti(0001) free surface after adding an alloying element, $E_{Mg/Ti-TM}^{\mathrm{int}erface}$ is the total energy of the Mg/Ti interface after adding an alloying element. Figure 8b illustrates the *W*_ad_ of the Mg(0001)/Ti(0001) interfaces after the introduction of alloying elements. The dashed line within the figure represents the magnitude of *W*_ad_ for the pure Mg(0001)/Ti(0001) interface. Generally, the larger the *W*_ad_ of the interface is, the stronger the cohesive strength of interface atoms is, whereas the opposite scenario indicates a weakening of cohesive strength. Further analysis reveals that, besides Gd, the doping of alloying elements such as Sc, V, Y, Zr, Nb, and Mo also leads to a slight increase in the *W*_ad_ of the Mg(0001)/Ti(0001) interface. This indicates that these alloying elements also have a strengthening effect on the cohesive strength of the interface to some extent.

#### 2.2.3. Electronic Structure

**Figure 9:**
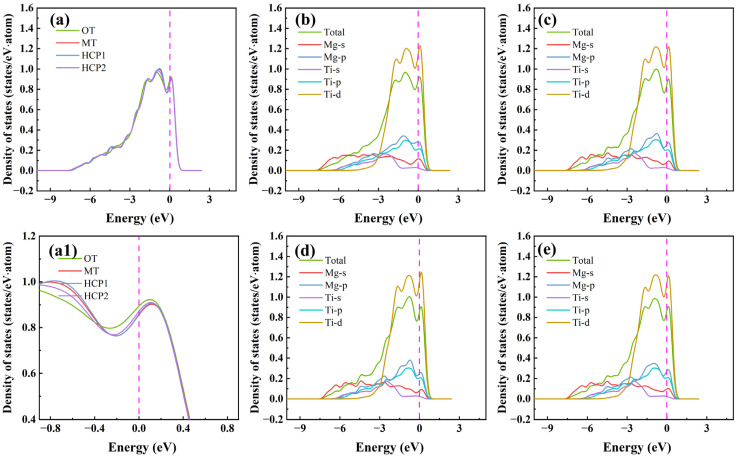
The total density of states (**a**) (where (**a1**) is a magnified view of (**a**) near the Fermi level) and partial density of states (**b**–**e**) with different interface configurations for the Mg(0001)/Ti(0001) interface. (**b**) OT configuration, (**c**) MT configuration, (**d**) HCP1 configuration, and (**e**) HCP2 configuration.

It utilizes first-principles methods to examine the electronic structure of the pure Mg/Ti interface and doping Mg/Ti interface configurations, allowing for a detailed exploration of atomic electron levels and revealing the fundamental nature of crystal structures. This capability is one of the key advantages of first-principles calculations, offering valuable insights for the advancement of materials design and engineering. The total and partial density of states for different interface configurations of the Mg(0001)/Ti(0001) are depicted in Figure 9. Evidently, based on the total density of states near the Fermi level (E_F_), all the Mg(0001)/Ti(0001) interface configurations exhibit metallic properties. The total density of states shows little variation across different configurations. Since the primary performance differences arise from variations in the electronic configurations near the Fermi level, the partial density of states from −9 eV to 3 eV can be plotted, as shown in Figure 9b–e. The density of states near E_F_ is predominantly influenced by Mg-*p* and Ti-*d* states. Notably, structural properties, such as cohesion, elastic constants, and interface energy, are primarily associated with the *d*-band within the Ti atoms. For the Mg/Ti interface with OT, MT, HCP1, and HCP2 configurations, it is evident that around the energy of −3 eV, there is a clear overlap between Mg-*s* and Ti-*p* orbitals, indicating the presence of *sp* hybridization. Moreover, to clearly see the difference in the density of states near the E_F_, we magnified the density of states in Figure 9a near the E_F_, as shown in Figure 9(a1). Noticeably, it is apparent that the total density of states near the E_F_ is lowest for the Mg(0001)/Ti(0001) interface with the HCP2 configuration, suggesting its highest stability. This correlates with the previously computed results for the *W*_ad_.

**Figure 10:**
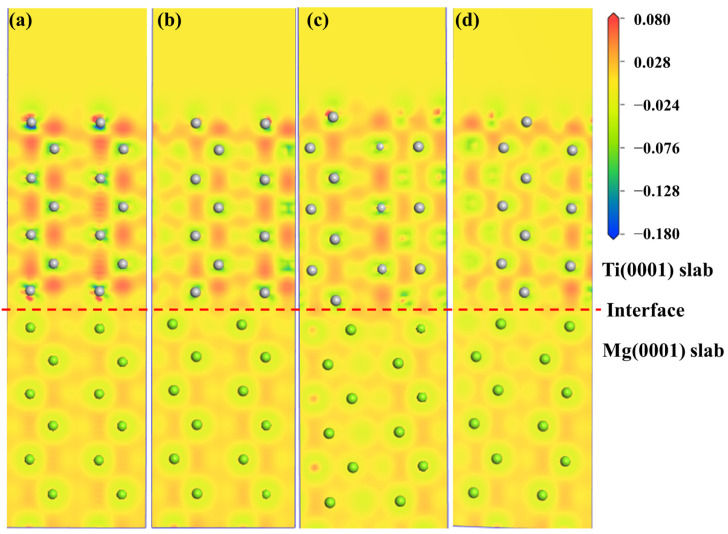
The charge density difference maps with different interface configurations for the Mg(0001)/Ti(0001) interface: (**a**) OT configuration, (**b**) MT configuration, (**c**) HCP1 configuration, and (**d**) HCP2 configuration, and the dashed lines indicate the Mg/Ti interface.

**Figure 11:**
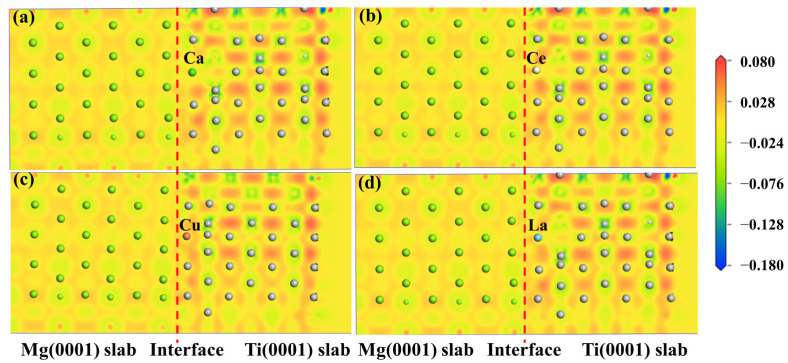
The charge density difference maps of Mg(0001)/Ti(0001) interface with different alloy elements doping the first layer of Ti atoms: (**a**) doping Ca atom, (**b**) doping Ce atom, (**c**) doping Cu atom, and (**d**) doping La atom, and the dashed lines indicate the Mg/Ti interface.

**Figure 12:**
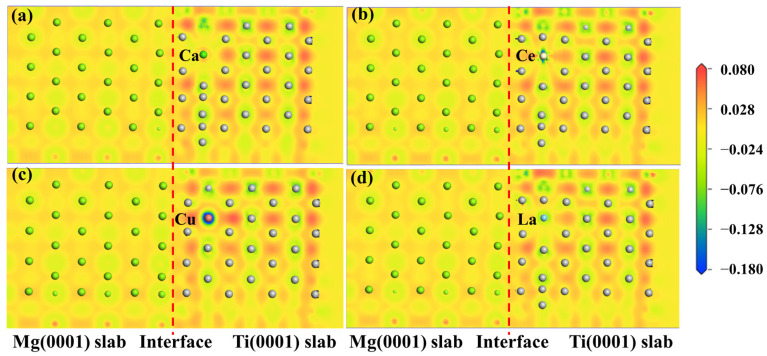
The charge density difference maps of Mg(0001)/Ti(0001) interface with different alloy elements doping the sub-surface layer of Ti atoms, (**a**) doping Ca atom, (**b**) doping Ce atom, (**c**) doping Cu atom, and (**d**) doping La atom, and the dashed lines indicate the Mg/Ti interface.

The electronic structure determines the mechanical properties of the Mg/Ti interfaces. To comprehend the microscopic mechanisms that govern the interface properties of Mg(0001)/Ti(0001), the interface’s charge density difference was plotted, as depicted in Figure 10. We can observe from Figure 10 that the degree of localization near Ti atoms is higher compared to that near Mg atoms, indicating a higher degree of delocalization near Mg atoms. Furthermore, among all interface configuration structures, the HCP2 interface exhibits a higher localization at the interface, indicating stronger metallic bonding between Mg and Ti and, thus, a more stable interface structure. Furthermore, to analyze the influence of different alloying elements on the interfacial segregation behavior of the Mg(0001)/Ti(0001) interface, several typical alloying elements (including Ca, La, Cu, and Ce) are chosen, and the charge density difference in these interface models after doping is analyzed. The specific results are depicted in Figure 11 and Figure 12. The red dashed line in Figure 11 and Figure 12 represents the interface position, where the section on the right side corresponds to the Ti(0001) layer, and the section on the left side corresponds to the Mg(0001) layer. Indeed, it is clear that the region surrounding Ti atoms is depicted in red, whereas the area surrounding Mg atoms is shown in blue, suggesting that Ti atoms gain electrons while Mg atoms lose electrons. Compared to the interface electronic structure without doping, the distribution of electron clouds changes when introducing different alloying elements to the interface. Additionally, it can be observed that the delocalization around Ca atoms is more significant than that around Ce, Cu, and La atoms, and there is partial overlap between the electron clouds of Mg and Ca atoms, indicating the formation of strong metallic bonds between Ca atoms and Ti or Mg, thereby enhancing the interface. However, the charge density difference plots of Mg(0001)/Ti(0001) interfaces with different alloying elements doped near the sub-surface Ti atoms are notably different from those doped near the surface Ti atoms. The electronic structure around the second layer of Ti atoms has undergone significant changes, indicating the formation of TM-Ti bonds. It can be observed that the area surrounding Cu atoms is encapsulated by blue spherical shapes, while the region around Ce atoms exhibits a symmetric distribution resembling “petals”, which is related to the waveforms associated with Ce atom’s *d* orbitals. Exactly during plastic deformation, the non-spherical distribution of electrons serves as a barrier, hindering the material’s plastic deformation and ultimately improving its mechanical properties. In all interfaces, electrons around the bulk Mg atoms transfer to the Mg atoms at the interface, weakening the metallic characteristics of Mg-Mg bonds and strengthening their ionic characteristics, leading to increased stability of the interface structure.

## 3. Computational Method Details

The first-principles calculations of the bulk and surface properties of Mg and Ti, as well as interface properties of Mg/Ti composites, were conducted using the density functional theory (DFT) within the framework of the Cambridge Serial Total Energy Package Code (CASTEP) [73]. Additionally, the segregation behavior of alloying elements Si, Ca, Sc, V, Cr, Mn, Fe, Cu, Zn, Y, Zr, Nb, Mo, Sn, La, Ce, Nd, and Gd at the interfaces was investigated systematically [65,74]. The interaction between valence electrons and ion cores was characterized using ultrasoft pseudopotentials (USPPs) [75]. For Mg, Ti, and the aforementioned alloying elements, their respective valence electron configurations were as: 2*p*^6^3*s*^2^, 3*s*^2^3*p*^6^3*d*^2^4*s*^2^, 3*s*^2^3*p*^2^, 3*s*^2^3*p*^6^4*s*^2^, 3*s*^2^3*p*^6^3*d*^1^4*s*^2^, 3*s*^2^3*p*^6^3*d*^3^4*s*^2^, 3*s*^2^3*p*^6^3*d*^5^4*s*^1^, 3*d*^5^4*s*^2^, 3*d*^6^4*s*^2^, 3*d*^10^4*s*^1^, 3*d*^10^4*s*^2^, 4*d*^1^5*s*^2^, 4*s*^2^4*p*^6^4*d*^2^5*s*^2^, 4*s*^2^4*p*^6^4*d*^4^5*s*^1^, 4*s*^2^4*p*^6^4*d*^5^5*s*^1^, 5*s*^2^5*p*^2^, 5*s*^2^5*p*^6^5*d*^1^6*s*^2^, 4*f*^1^5*s*^2^5*p*^6^5*d*^1^6*s*^2^, 4*f*^4^5*s*^2^5*p*^6^6*s*^2^ and 4*f*^7^5*s*^2^5*p*^6^5*d*^1^6*s*^2^, respectively. To achieve convergence and ensure computational accuracy, the maximum kinetic energy cutoff for the expansion of plane waves in reciprocal space was established at 450 eV. For bulk Mg and Ti, a 9 × 9 × 9 grid of *k* points was chosen for sampling the first Brillouin zone (BZ). For the Mg(0001) and Ti(0001) surfaces, the *k* point was set as 7 × 7 × 1 to ensure the convergence. The Mg/Ti interface models with different atomic stacking sequences were built using a 2 × 2 supercell model to guarantee periodic boundary conditions, which contained 56 Mg and 56 Ti atoms, and the interface models had 4 × 2 × 1 *k* points. Moreover, the Mg(0001) and Ti(0001) surface slab models with a 2 × 2 supercell model consisting of seven atomic layers were adopted to establish the Mg(0001)/Ti(0001) interface models with different atomic stacking sequences according to our previous work [76]. Additionally, a vacuum region of 15 Å was employed to eliminate interactions between atoms at the interface. In addition, to achieve equilibrium in the bulk, surface, and interface structures, the Broyden–Fletcher–Goldfarb–Shanno (BFGS) algorithm was employed [77,78]. Additionally, this was a quasi-Newton method, which iteratively solves for the minimum point of an objective function by gradually constructing an approximation of the inverse of the Hessian matrix. The exchange-correlation function was modeled using the Perdew–Burke–Ernzerhof (PBE) within a generalized gradient approximation (GGA) [79] to calculate the bulk, surface, and interface properties. The convergence criteria for the total energy, atomic forces, and displacements in each crystal and interface structure were established at 1 × 10^−6^ eV, 0.01 eV/Å, and 0.001 Å, respectively.

## 4. Conclusions

This study utilizes first-principles calculations based on density functional theory to explore the interface stability and the interfacial segregation behavior of alloying elements at the interface of the Mg/Ti interface. Through an in-depth analysis of interface electronic properties, the stability of the interface and the segregation behavior of elements at the interface are examined. The main conclusions of this work are as follows:

(1) The calculated phonon spectra of Mg and Ti indicate that both are dynamically stable phases. Additionally, criteria for mechanical stability also suggest that they are mechanically stable phases. The calculated elastic properties results show that Ti exhibits the largest C_11_ value of approximately 205.9 GPa and Young’s modulus of around 153.4 GPa, indicating that Ti can effectively enhance the modulus of Mg alloys.

(2) First-principles calculations determined that the optimal interface distance for the Mg/Ti interface configurations is approximately 2.5 Å. They are using seven-layer Mg(0001) surface and seven-layer Ti(0001) surface slab models to build Mg(0001)/Ti(0001) interface configurations with OT, MT, HCP1, and HCP2 atomic stacking interface configurations. Based on the results of interface adhesion work and electronic structure information, it can be concluded that the Mg(0001)/Ti(0001) interface with HCP2 configuration has the best stability.

(3) The segregation calculations for eighteen alloying elements near the Mg(0001)/Ti(0001) interface indicate that Gd atoms, when doped into the first layer of Ti atoms, result in the lowest heat of segregation of −5.83 eV, suggesting that Gd is the most prone to segregation. Also, alloying elements such as Si, Sn, and Nd tend to segregate at the interface. Conversely, alloying elements like Ca, La, Cr, and V are less likely to segregate. These findings are further supported by electronic structure information.

## Data Availability

Data are contained within the article.
